# Socio-Economic Determinants of Contraceptive Use among Married Women in the Addis Urban Health and Demographic Surveillance System (Addis-HDSS) in Ethiopia

**DOI:** 10.4314/ejhs.v34i2.8S

**Published:** 2024-12

**Authors:** Hanna Yemane Berhane, Semira Abdelmenan, Firehiwot Workneh, Dagmawit Tewahido, Tigest Shifraw, Kalkidan Yibeltal, Workagegnhu Tarekegn, Nebiyou Fasil, Dongqing Wang, Uttara Partap, Wafaie Fawzi, Meaza Demissie, Alemayehu Worku, Yemane Berhane

**Affiliations:** 1 Nutrition and Behavioral Sciences Department, Addis Continental Institute of Public Health, Addis Ababa, Ethiopia; 2 Epidemiology and Biostatistics Department, Addis Continental Institute of Public Health, Addis Ababa, Ethiopia; 3 Reproductive Health Department, Addis Continental Institute of Public Health, Addis Ababa, Ethiopia; 4 Department of Global Health and Health Policy, Addis Continental Institute of Public Health, Addis Ababa, Ethiopia; 5 Department of Global and Community Health, College of Public Health, George Mason University, Virginia, USA; 6 Department of Global Health and Population, Harvard T. H. Chan School of Public Health, Boston, Massachusetts, USA; 7 School of Public Health, Addis Ababa University, Addis Ababa, Ethiopia

**Keywords:** Socio-Economic Determinants, Contraceptive Use, Married Women, Addis Ababa, Urban Health Demographic Surveillance System, Addis-HDSS, Ethiopia

## Abstract

**Background:**

Contraceptives are essential for protecting women's sexual and reproductive health, as well as for reducing maternal and infant morbidity and mortality. This study aimed to identify socioeconomic factors associated with contraceptive use among married women of childbearing age in Ethiopia.

**Methods:**

We analysed cross-sectional data from 15,499 married women aged 18-49 years, collected as part of the Urban Health and Demographic Surveillance System (HDSS) in Addis Ababa. Trained interviewers conducted face-to-face interviews using structured, pre-tested questionnaires. Bivariable and multivariable logistic regression models were used to calculate adjusted odds ratios (AOR) with 95% confidence intervals (CIs) to assess associations between socioeconomic factors and contraceptive use.

**Results:**

The overall prevalence of contraceptive use among married women was 53.5% (95% CI: 52.7-54.4%). Women with larger family sizes (>6) (AOR: 2.1; 95% CI: 1.5-2.8), excellent self-reported health (AOR: 1.6; 95% CI: 1.3-1.9), and those in households with adequate income to meet basic needs (AOR: 1.29; 95% CI: 1.11-1.48) were more likely to use contraceptives. Women over 40 years old (AOR: 0.21; 95% CI: 0.18-0.25) and those living in female-headed households (AOR: 0.68; 95% CI: 0.61-0.76) had lower odds of using contraception.

**Conclusion:**

About half of urban married women of reproductive age reported using contraceptives. Factors associated with higher contraceptive use included better self-reported health and larger family sizes. Further research is needed to understand the complex dynamics of contraceptive use in low-income urban settings.

## Introduction

Contraceptives enable individuals and couples to control the number and spacing of their children while promoting fundamental human rights, such as the right to life, liberty, and education (1). Proper use of contraceptives offers numerous benefits, including the prevention of unintended pregnancies, reduced psychological strain, and decreased morbidity and mortality. Additionally, contraceptive use contributes to broader community health by lowering rates of low birth weight, unwanted pregnancies, and health costs, thereby freeing up resources for other essential areas like education (2–6).

Ensuring universal access to family planning services is one of the key strategies to achieving sustainable development (7). However, global estimates show family planning needs satisfied by contraceptives have stagnated in the past 5 years at 77% (1), with vast disparities across regions and population groups (8). In low- and middle-income countries, despite the benefits, contraception remains underutilized. For instance, a metaanalysis found that the pooled postpartum contraceptive prevalence rate was only 41% (9). The results of another study examining demographic and health surveys of 37 countries in sub-Saharan Africa revealed that the prevalence of contraceptive use ranged from 3.5% to 49.7%, with an overall average of 22% (10). Consequently, high rates of unintended pregnancies and abortion persist in low-income countries, contributing to avoidable maternal deaths (11–13).

The use of contraceptives is influenced by a variety of socioeconomic factors, including family size (10,14), age (14,15), women's income (10), household wealth (16,17), women's education level (14–16,18), and media access (15,17). Generally, women with a better status in these and other socioeconomic factors are likely to better utilize contraceptives. The role of each factor varies depending on the context. In urban areas, where inequalities and population size are increasing exponentially, understanding the social determinants of contraceptive use is crucial for developing effective interventions. This study examines the socioeconomic factors associated with contraceptive use among married women of childbearing age in Addis Ababa, Ethiopia.

## Methods

**Study design and setting**: The Addis Ababa Urban Health and Demographic Surveillance System (Addis-HDSS) was established in 2022 to provide a platform for community-based research and support the generation of high-quality health data. For this study, data were collected from Yeka, one of Addis Ababa's largest and oldest sub-cities, which is home to two government hospitals, ten health centers, and 84 private health services. The first round of the survey, conducted from December 2022 to January 2023, included six of the ten woredas (districts) and 240 enumeration areas.

**Sampling and data collection**: The Addis-HDSS surveyed all eligible households within the catchment area using a complete census. Structured questionnaires were administered to residents in Amharic, Ethiopia's national language, using tablet devices. In every household, trained data collectors gathered general information as well as specific details targeting women of reproductive age (18-49 years), inquiring about pregnancy status, intentions regarding pregnancy, and contraceptive use. Of the 35,233 women in this age group, 15,499 were married, which constituted the study sample.

**Measurement**: Contraceptive use was measured by calculating the proportion of married women within the age group 18-49 years who use any type of contraceptive, women who reported to be pregnant at the time of the survey were excluded (n=984). All others were then asked, “Do you currently use any kind of contraceptive?” Women who responded “yes” were considered users. Currently married women who responded “No” to the questions “Do you intend to get pregnant in the next 12 months” and “Do you currently use any kind of contraceptive” were considered to have unmet needs.

Sociodemographic variables included the women's educational attainment (categorized as no formal education, primary, high school, and college/university), age (grouped into 18-24, 25-29, 30-34, 35-39, and 40+ years), and perceived health status (rated as poor, fair, good, very good, or excellent). Family size was categorized as 2 or fewer, 3-4, 5-6, and more than 6. Household income adequacy was assessed by asking whether the household's monthly income was sufficient to meet basic needs (options ranged from “not at all” to “adequately”). A household was classified as female-headed if the respondent or another woman was identified as the head of the household.

**Statistical analysis**: Descriptive statistics (means, standard deviations, and proportions) were used to summarize the data. Bivariable logistic regression was employed to assess associations between each socioeconomic factor and contraceptive use. Variables with a p-value of less than 0.20 in the bivariable analysis were included in multivariable models to adjust for potential confounders. Adjusted odds ratios (AOR) and 95% confidence intervals (CIs) were reported. Statistical analysis was conducted using STATA version 14.

**Ethical considerations**: The study protocol was approved by the Ethical Review Committee of Addis Continental Institute of Public Health (Reference no. ACIPH/IRB/003/2022). Written informed consent was obtained from all participants, and data was anonymized to ensure confidentiality.

## Results

**Sociodemographic characteristics**: The study included 15,499 married women of reproductive age from the Addis-HDSS site. The mean age of the women was 33.1 ± 7 years. A significant portion (93.2%) had received formal education, with 36% having a college or university level education. Approximately 57% of the women lived in shared housing or compounds. Half (52%) were engaged in income-generating activities or were employed. Notably, 20% of the women reported that their household's monthly income was insufficient to meet basic needs (Table 1).

**Table 1:** Socio-economic characteristics of the women in the reproductive age group (18-49 years) in the Addis-HDSS, Ethiopia, 2023; (n=154)

Characteristics	Number	%
Age (years) (mean ± standard deviation)	33.1±7	
Age group (years)		
<25	1522	9.82
25-29	3935	25.4
30-34	3624	23.4
35-39	3275	21.1
40-49	3143	20.3
Attended formal education		
No	1049	6.8
Yes	14450	93.2
Grade Level		
No formal education	1049	6.7
Primary education (≤ grade 8)	4312	27.8
Secondary education (grades 9-12)	4936	31.9
College/university	5202	33.6
Employment status		
Not employed	7404	47.8
Currently employed	8,095	52.2
Female-headed family		
No	13725	88.5
Yes	1774	11.5
Housing unit they live in		
House with a separate compound	3880	25.0
House with no compound	1277	8.2
house/room in a shared compound	8885	57.3
Apartment unit/condominium	1272	8.2
A room in a shared	165	1.1
apartment/condo		
shared room/other	20	0.1
Regular monthly family income covers basic needs		
not at all	3165	20.4
Yes, minimally	5239	33.8
Yes, moderately	4913	31.7
Yes, adequately	2182	14.1
Health Insurance		
No	10738	69.3
Yes	4761	30.7

**Self-reported health, pregnancy, and contraceptive use**: In terms of self-reported health, most women rated their health as good (26.8%), very good (48.4%), or excellent (21.0%). Ninety-three percent of the women were not currently pregnant, and of those, 17% expressed an intention to become pregnant. The overall prevalence of contraceptive use was 53.5% (95% CI: 52.7–54.4%), and the unmet need for contraception was 42.7% (95% CI: 41.8–43.6%) (Table 2).

**Table 2:** Self-reported health status and contraceptive use among married women in reproductive age group (18-49 years) in the Addis-HDSS, Ethiopia, 2023

Characteristics	Number	%
Self-reported health status (n=14,041)		
Poor, fair	532	3.8
Good	3764	26.8
Very Good	6802	48.4
Excellent	2943	21.0
Current pregnancy (n= 15,499)		
No	14350	92.6
Yes	984	6.4
Don't Know	165	1.1
Intention to get pregnant (n=14, 515)		
No	12045	83.0
Yes	2470	17.0
Current use of contraceptives (n=14, 515)		
No	6742	46.5
Yes	7773	53.5
Unmet need for contraceptives (n=12045)		
No	6904	57.3
Yes	5141	42.7

**Determinants of contraceptive utilization**: Contraceptive use was positively associated with larger family sizes, better self-reported health, and the ability of the household to meet basic needs. Women with a family size of six or more were more likely to use contraception than those with a family size of two or fewer (AOR: 2.08; 95% CI: 1.53–2.83). Women who rated their health as good, very good, or excellent were at least 1.5 times more likely to use contraception compared to those who rated their health as poor or fair. Similarly, compared to women from households that believed their income was insufficient to meet basic needs, those who believed they could meet their basic needs (even minimally) were more than 1.2 times more likely to use contraception (Table 3).

**Table 3:** Association of socio-economic variables with contraceptive utilization among married women in the reproductive age group (18-49 years) in the Addis-HDSS, Ethiopia, 2023

Characteristics	Contraceptive use			

	Yes	NO		

	Number (%)	Number (%)	COR (95% CI) ^a^	AOR (95% CI) ^b^
Female-headed family				
Male headed family	7043 (54.9)	5793 (45.1)	Reference	Reference
Female-headed family	730 (43.5)	949(56.5)	**0.63 (0.57-0.70)**	**0.68 (0.61-0.76)**
Health Insurance				
Does not have insurance	5525 (55.2)	4482 (44.8)	Reference	Reference
Has health insurance	2248 (49.9)	2260 (50.1)	**0.87 (0.75- 0.87)**	0.93 (0.85- 1.02)
Employment status				
Not employed	3861 (56.2)	3013 (43.8)	Reference	Reference
Employed	3912 (51.2)	3729 (48.8)	**0.82 (0.77- 0.87)**	0.98 (0.91- 1.03)
Women's education				
No formal education	517 (51.6)	486 (48.5)	Reference	Reference
Primary school (grades 0-8)	2365 (58.5)	1676 (41.47)	**1.33 (1.15- 1.52)**	1.09 (0.94-1.28)
Secondary school (grades 9-12)	2524 (54.3)	2125 (45.7)	1.12 (0.97- 1.28)	1.05 (0.90-1.22)
College/university	2367 (49.1)	2455 (50.9)	0.91 (0.80-1.04)	0.87 (0.74-1.03)
Age group				
<25 years	894(66.9)	442(33.1)	Reference	Reference
25-29 years	2217(62.9)	1308(37.1)	**0.84(0.73-0.96)**	0.74 (0.64-0.85) *
30-34 years	2002(59.3)	1373(40.7)	**0.72(0.63-0.82)**	0.63 (0.54-0.73) *
35-40 years	1618(51.3)	1538(48.7)	**0.52(0.46-0.59)**	0.45 (0.38-0.52) *
≥ 40 years	1042(33.4)	2081 (66.6)	**0.25(0.22-0.28)**	0.21 (0.18-0.25) *
Family size				
≤ 2	623(41.9)	864(58.1)	Reference	Reference
3 to 4	4324(56.6)	3321(43.4)	**1.81(1.61-2.02)**	2.17(1.91-2.45 *
5 to 6	2341(53.9)	2005(46.1)	**1.62(1.44-1.82)**	2.75(2.39-3.16) *
≥ 7	200(56.5)	260(43.5)	1.07(0.86-1.32)	2.08(1.53-2.83) *
Perceived health status				
Poor or fair	210 (40.7)	306 (59.3)	Reference	Reference
Good	1897(53.4)	1657(46.6)	**1.67(1.38-2.01)**	1.46(1.19-1.78) *
Very good	3489(55.1)	2847(44.9)	**1.79(1.49-2.14)**	1.46(1.20-1.79) *
Excellent	1538(56.2)	1200(43.8)	**1.87(1.54-2.27)**	1.57(1.28-1.93) *
Does regular monthly family income cover basic needs				
No, not at all	1570 (52.8)	1402 (47.2)	Reference	Reference
Yes, minimally	2696 (54.9)	2209 (45.1)	**1.09 (0.99-1.19)**	**1.22 (1.1-1.35)**
Yes, moderately	2533 (55.3)	2047 (44.7)	**1.12 (1.01-1.21)**	**1.38 (1.23-1.53)**
Yes, adequately	974 (47.3)	1084 (52.7)	**0.80 (0.72-0.89)**	**1.29 (1.11-1.48)**

Abbreviations: OR odds ratio; CI, confidence interval; COR, crude odds ratio; AOR, adjusted odds ratio

* significance level of <0.05

a Model 1= unadjusted

b Model 2=adjusted for household wealth quintile

Contraceptive use was inversely associated with age; women aged 40 years or older were significantly less likely to use contraception compared to those under 25 (AOR: 0.20; 95% CI: 0.17–0.24). The likelihood of contraceptive use was also lower in female-headed households (AOR: 0.68; 95% CI: 0.61–0.76) (Table 3).

## Discussion

The study found that 53.5% of married women of reproductive age in Addis Ababa used contraceptives. The likelihood of using contraception was higher among women with larger family sizes, better self-reported health, and those who believed their household income could meet basic needs. Conversely, contraceptive use was lower among older women and those living in female-headed households.

Nearly half of the married women in our study did not use contraception, which aligns with findings from the 2019 Ethiopian Demographic and Health Survey (EDHS), where 48% of currently married women in urban areas used modern contraceptives (19). Over the past 15 years, the national contraceptive use rate has tripled, from 14% in 2005 to 41% in 2019(19). However, it remains concerning that 4 out of 10 married women of reproductive age who do not wish to become pregnant are not using contraceptives. Although the result from this study is slightly higher, other national and regional studies have also found that approximately a third of the women have an unmet need for modern contraceptives, and higher unintended pregnancies (20,21). This highlights the need to intensify national efforts to increase contraceptive utilization among married women.

In our study, women from households that believe their monthly income could cover their basic needs are more likely to use contraception. To the best of our knowledge, this perception measure has not been used in other studies; but the findings are in line with earlier studies that showed that women in wealthier households were more likely to use contraception. Gebre et al. found that women in the wealthiest households were 3.5 times more likely to use contraceptives compared to those in the poorest households (17). Other studies have also reported similar findings, indicating that as wealth increases, contraceptive utilization also increases (22,23). Studies from India and Malawi have also attested to this, indicating that contraceptive use increases in the richest wealth quintile (24,25). A potential explanation for our findings is perhaps the women in the wealthiest households have better access to health care and media sources that equip them with the information they need to make informed decisions about themselves and their families.

Contraceptive use was inversely associated with age, with older women (40 years and above) less likely to use contraception compared to younger women. This is consistent with findings from a nationally representative study, which reported that younger women (ages 15-19) are nearly twice as likely to use contraceptives as women aged 40 and above (17). However, other regional studies have shown contradictory results, with middle-aged women being more likely to use contraceptives (14). Another study from the Eastern part, reports that younger women were less likely to use contraceptives as compared to their older counterparts 35 years and above (26). Some of these inconsistencies may be related to cultural values. In some cultures, women are expected to have children immediately after getting married. However, in urban areas, women may delay pregnancy due to financial constraints until they can provide for their families. According to the EDHS 2019, the median age at first birth for urban women is nearly two years later than for rural women (22). Furthermore, we did not account for early menopause in this study, which could explain why older women aged 40 and up do not use contraception.

Women living in female-headed households were less likely to use contraceptives, which aligns with findings from a study across 21 sub-Saharan African countries (27). Other studies have also found similar results indicating lower contraceptive use among female-headed households (28,29), which could be attributed to lower sexual activity in female-headed households where partners may not be present for various reasons including employment opportunities. Another potential explanation could be social norms that hinder women from accessing reproductive health services without their partners.

Larger family size and better health status were positively associated with contraceptive use. Women with six or more children were twice as likely to use contraception compared to those with fewer children. This finding is in line with earlier studies (17,22,23) and could potentially be pointing to the fact that women who have reached their desired family size or are satisfied with their children's sex composition are more likely to use contraceptives. Some research has indicated that in cultures where larger family sizes are valued, there tends to be a decrease in the use of contraceptives (30,31). Additionally, women who rated their health as good or excellent were more likely to use contraception, perhaps because individuals with poor health might not recognize their susceptibility to pregnancy, possibly due to the impact of their health on sexual activity and ability to conceive (32). Fear of contraceptives further worsening their health may also be a contributing factor. On the other hand, healthier women are more proactive about managing their reproductive health.

While the study has a large sample size and benefits from the Addis-HDSS platform, it also has limitations, such as the inability to account for partners' educational levels and support, which may influence contraceptive use. Additionally, the self-reported nature of contraceptive use raises the possibility of social desirability bias, where participants may over- or under-report their contraceptive use based on perceived social acceptability.

In conclusion, less than half of married women of reproductive age in Addis Ababa use contraception. To increase contraceptive use, it is crucial to address the complex determinants and develop targeted programs for key groups, especially those with lower socioeconomic status and younger women, to maximize the effectiveness of family planning interventions.
